# A Looming Storm on the Horizon

**DOI:** 10.3201/eid3005.AC3005

**Published:** 2024-05

**Authors:** Byron Breedlove

**Keywords:** Crimean-Congo hemorrhagic fever, viruses, art science connection, emerging infectious diseases, art and medicine, about the cover, Vladimir Orlovsky, Ukraine, tick-borne virus, *Hyalomma* *spp*. ticks, a looming storm on the horizon

**Figure:**
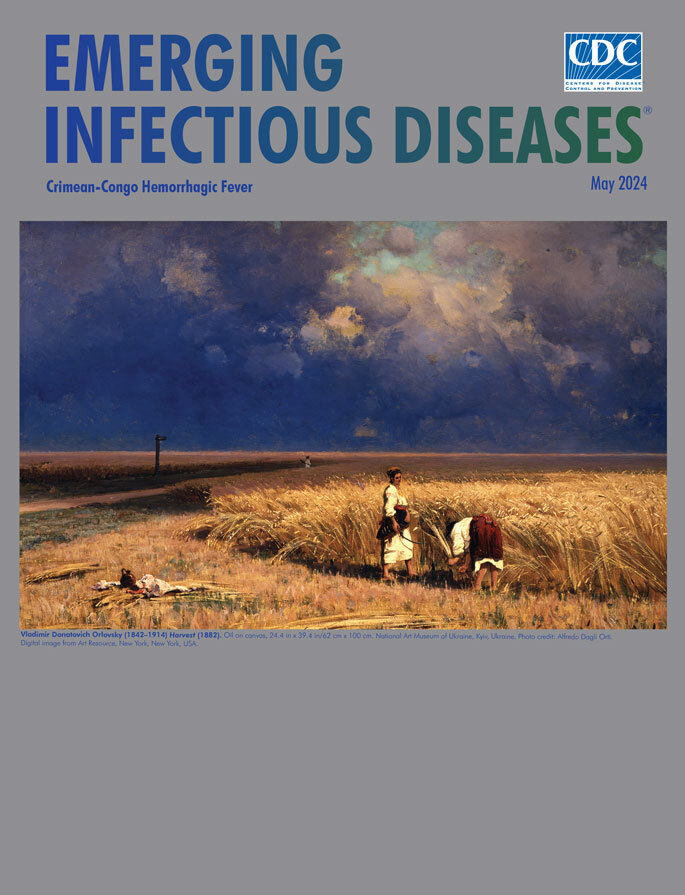
**Vladimir Donatovich Orlovsky (1842–1914) *Harvest* (1882).** Oil on canvas, 24.4 in x 39.4 in/62 cm x 100 cm. National Art Museum of Ukraine, Kyiv, Ukraine. Photo credit: Alfredo Dagli Orti. Digital image from Art Resource, New York, New York, USA.

Ukrainian realist painter Vladimir Orlovsky was born in Kiev (now Kyiv), Ukraine, where he received his early artistic training. In 1861, he attended the Saint Petersburg Academy of Fine Arts. After graduating in 1868, Orlovsky earned a medal of recognition and monetary award for a series of paintings of Crimean landscapes, enabling him to travel and paint throughout Europe for the next 3 years. He later joined the Saint Petersburg Academy faculty and in 1878 was named a professor of art. His work was popular among the aristocratic class, including the Emperor of Russia, Alexander III.

According to the *Encyclopedia of Ukraine*, “In 1886 he returned to Ukraine, where he taught at the Kyiv Drawing School and helped found the Kyiv Art School. As Orlovsky gradually freed himself from academism, his works acquired a more natural composition, more confident lines, and a finer coloration.” *Harvest*, this month’s cover image, is one among “his famous depictions of the Ukrainian and Crimean countryside.”

In *Harvest*, Orlovsky depicted workers on the Ukrainian steppe cutting grain with scythes. In the foreground, one worker bends and grasps the stalks as the other looks across the field. To their left are a brown jug and perhaps remains of a midday meal. The uncut grain twitches and bends, pressed by winds from the rainstorm swelling over the plains. A third person walking in the distance and the remote horizon revealed as a thin strip of light define the scale of the steppes. A maelstrom of purplish clouds laden with rain roils above the fields save an open space of blue sky tinged with white clouds that illuminates the lower third of the canvas. Despite the pending storm, the workers seem untroubled and intent on their task.

An outbreak of a tickborne hemorrhagic disease documented in Crimea in 1944 may have occurred in a setting similar to the one in Orlovsky’s painting. That disease, now called Crimean-Congo hemorrhagic fever (CCHF), is caused by infection with a tickborne *Nairovirus,* Crimean-Congo hemorrhagic fever virus (CCHFV), in the family Bunyaviridae. In an article in *Antiviral Research*, Dennis A. Bente and coauthors wrote that researchers investigating that outbreak in Crimea “were quickly able to link cases of the new disease to tick exposure. They noted that, because large areas of cultivated land had been abandoned during the German occupation, the population of hares and other wild hosts of *Hyalomma* ticks had increased, and soldiers and farm workers engaged in restoring agricultural production were suffering large numbers of tick bites.”

Originally known as Crimean hemorrhagic fever, the disease acquired its current hyphenated name after identical symptoms were reported in the Democratic Republic of the Congo in 1969. It has been around much longer, of course, and researcher Chris A. Whitehouse reported that a hemorrhagic disease described by a 12th-century physician in the region today called Tadzhikistan was probably CCHF. As Whitehouse noted, CCHF is indicated by “the presence of blood in the urine, rectum, gums, vomitus, sputum, and abdominal cavity.”

According to the World Health Organization (WHO), hosts for the ticks include many wild animals and domestic animals such as cattle, sheep, and goats; most cases of CCHF among humans have occurred among people working with livestock, including those preparing carcasses of infected animals. WHO also states, “Human-to-human transmission can occur resulting from close contact with the blood, secretions, organs or other bodily fluids of infected persons.” Bente and his coauthors stated that CCHF “is the most widespread tick-borne viral infection of humans, occurring across a vast area from western China through southern Asia and the Middle East to southeastern Europe and throughout most of Africa.” WHO considers CCHF a high-priority disease. The case-fatality rate is as high as 40%, and the disease is both difficult to prevent and challenging to treat.

Several articles in this issue address CCHF, including a three-part series, Crimean-Congo Hemorrhagic Fever Virus for Clinicians, by M.G. Frank et al., which addresses a trilogy of topics: diagnosis, clinical management, and therapeutics; virology, pathogenesis, and pathology; and epidemiology, clinical manifestations, and prevention. The authors report “lack of licensed effective therapeutic and prophylactic drugs, gaps in our understanding of CCHFV pathogenesis and immunology, and slow progression in development of CCHF medical countermeasures.”

As temperatures increase around the world, could CCHF become the next storm brewing on public health horizon, like the dark clouds in Orlovsky’s *Harvest*? CDC researcher Jessica Spengler and her coauthors wrote in a 2019 article, “Without question, frequency of disease reporting is increasing. Whether this represents expansion to new regions or changes to existing areas of sporadic circulation will continue to be challenging to differentiate. This rise could be due to increased awareness, but awareness is unlikely to be the only contributor.”
